# Screening is not always healthy: an ethical analysis of health screening packages in Singapore

**DOI:** 10.1186/s12910-022-00798-5

**Published:** 2022-06-07

**Authors:** Sarah Ee Fang Yong, Mee Lian Wong, Teck Chuan Voo

**Affiliations:** 1 Saw Swee Hock School of Public Health, National University of Singapore, Tahir Foundation Building (MD1), 12 Science Drive 2, Singapore, 117549 Singapore; 2 Centre for Biomedical Ethics, National University of Singapore, Yong Loo Lin School of Medicine, Block MD11, Clinical Research Centre, #02-03, 10 Medical Drive, Singapore, 117597 Singapore

**Keywords:** Health screening ethics, Health screening packages, Executive physical, Screening tests, Health screening policy

## Abstract

**Background:**

Health screening is undertaken to identify individuals who are deemed at higher risk of disease for further diagnostic testing so that they may possibly benefit from interventions to modify the natural course of disease. In Singapore, screening tests are widely available in the form of a package, which bundles multiple tests in one session and commonly includes non-recommended tests. There are various ethical issues associated with such testing as they may not be clinically appropriate and can result in more harm than benefit. This article describes the practice of health screening packages, identifies the ethical issues arising from such packages and discusses the implications of these ethical issues on policy and practice of screening in Singapore.

**Methods:**

A content analysis of the websites of providers offering general health screening packages to individuals was conducted. A total of 14 health screening package providers were analysed for how packages were conducted and promoted, how clinically appropriate screening tests were, and the price range and composition of screening packages. A normative ethical analysis based on the four principles approach of beneficence, non-maleficence, autonomy and justice in biomedical ethics was used.

**Results:**

Twelve of the 14 providers included non-recommended tests such as tumour markers, treadmill stress tests and MRI scans in their general health screening packages. Package prices ranged from S$26 to S$10,561, with providers charging higher when more tests were included. Health screening packages were broadly conducted in three stages: (1) the offer and selection of a health screening package; (2) medical assessment and performance of screening tests; (3) a post-screening review. While material provided by all providers was factual, there was no information on the potential risks or harms of screening.

**Conclusion:**

Several ethical issues were identified that should be addressed with regard to health screening packages in Singapore. A key issue was the information gap between providers and patients, which may result in patients undergoing inappropriate testing that may be more harmful than beneficial. Health screening packages can stimulate unnecessary demand for healthcare and contribute to an inequitable distribution of healthcare resources.

## Background

Health screening is commonly undertaken to identify individuals who are deemed at higher risk for disease for further diagnostic testing so that they may possibly benefit from interventions to modify the natural course of disease [1–3]. Different tests have varying levels of scientific evidence and cost effectiveness recommending their use for screening [4]. Some tests are deemed to have a net population benefit and are made available through national programmes, such as diabetes screening [5, 6]. For other tests, their use as a screening tool is contentious as harms may outweigh benefits or there is no evidence for use in an individual without risk factors. Examples include tumour markers and an MRI prostate [4]. The practice of screening between and within countries varies widely, in large part dependent on how its healthcare system is structured and the prevailing health conditions affecting the population.

Screening is widely available in Singapore in the form of health screening packages (HSPs). The practice of HSPs is not limited to Singapore and is referred to by various names, including “executive physicals” in the United States, “master health check-up” in India or “health measurements, observations and tests (MOT)” in the United Kingdom [4, 7–9]. A HSP refers to a set of screening tests usually performed in one session; HSPs frequently bundle tests recommended for population screening and those which are not [4]. Screening, particularly the use of non-recommended tests, can result in false positives or overdiagnosis, both of which in turn can lead to more invasive testing and treatments with unclear or detrimental health effects. Aside from incurring unnecessary financial costs, it can trigger undue anxiety and psychological distress. Such packages may promote an overuse of healthcare resources for investigations that are neither cost-effective nor evidence-based [7].

### How screening packages developed

HSPs originated from the United States and are now common in other countries, including Australia, the United Kingdom, Thailand, India and Singapore [4, 9–13]. HSPs are often available as part of a suite of corporate benefits, and function as both a company branding exercise and as an investment into workplace productivity [14, 15]. While HSPs were initially targeted at corporate clientele, they have also been marketed to individuals [16].

The flourishing of HSPs has taken place within the context of three broader developments occurring at the global and local level: (1) the corporatisation of healthcare with its emphasis on cost efficiency; (2) an increasing shift in focus from treatment to prevention amidst an increasing burden from noncommunicable diseases on healthcare systems; (3) and, advances in technology that detect “abnormalities” at increasingly lower thresholds (which may not otherwise cause morbidity or death) [17–20]. The value proposition of HSPs seems to dovetail with these developments as they offer an expanding pool of would-be “patients” an opportunity for early diagnosis and treatment, and serve as a new market which healthcare organisations can tap on to generate revenue.

### Features of health screening packages (HSPs)

The promotion and delivery of screening packages can vary across countries due to differences in healthcare systems such as insurance coverage and provider factors such as whether they receive government funding. Points of variation include whether the HSP consists of a medical assessment by a doctor or a post-screening consult to review results [8, 21]. Nonetheless, HSPs share a set of features, which are: (1) a “comprehensive” battery of tests; (2) their market-oriented pricing; (3) their commodification as a healthcare service.

#### “Comprehensive” battery of tests

There are no “standard” tests that constitute a HSP. Rather, what appears to be consistent is the extensive number of tests offered in a myriad number of permutations. Packages often include a range of investigation modalities (e.g. blood, urine, imaging) that screen for a wide variety of conditions (e.g. cancer, cardiovascular, infections). Alongside tests actually recommended for screening in the general population, “unsuitable” tests lacking evidence for use in screening or which are suitable for only selected subpopulations are often included [16]. More complex and higher-risk investigations have also been offered, exposing asymptomatic individuals to avoidable harms, such as large doses of radiation in CT scans [9].

#### Market-oriented pricing

Most providers offer a selection of packages, charging higher if more tests are included. Despite the sometimes exorbitant price tag, consumers are willing to pay for these “comprehensive” packages likely because of the mistaken assumption that more tests equate to a better screening package [7]. Leading healthcare institutions, such as the Mayo Clinic and the Johns Hopkins Hospital in the United States, also market these packages, which may persuade individuals to undergo these tests due to the perception that they are of good quality and value [7, 16].

#### Commodification as a healthcare service

Although screening was originally conceived as a strategy to improve population health, the bundling of tests in a package transformed them into a new revenue stream to maximise monetary gains [1, 7]. Packages are often marketed as an efficient way to consolidate screenings and are conducted in a luxurious environment [7]. Health screening has been re-designed as a service that can be bought to improve health, with buyers choosing the package they want and adding on tests for aspects of health that they are interested in.

### Literature review

Although health screening is among the most widely debated topics in bioethics, there has been little discussion focused on HSPs. While there is an extensive literature on the ethics of screening, it would be beyond the scope of the paper to provide a comprehensive review. This section is organised into four parts, with the first three providing an overview of themes in the literature most relevant to HSPs, namely: (1) the external pressure for individuals to undergo screening; (2) the inherent tension between individual interests and public benefit in screening; (3) and, the understanding that screening is not “risk-free” and potentially harmful. The fourth part will discuss key findings in the literature on the practice of HSPs.

#### External pressure for individuals to undergo screening

In contrast to most clinical services which are sought when a patient presents with a problem, screening is undertaken by an individual without symptoms of the disease(s) concerned. The prompt to get screened often comes from health authorities, including doctors, so there is often “outside” pressure for individuals to undergo screening which may be detrimental to autonomy, an aspect of which is freedom from controlling influences [3]. Although commercial interests are not usually discussed in the context of screening, they have become increasingly influential. There is evidence that financial relationships between industry and doctors can unduly influence professional judgment and lead to reduced focus on a patient’s best interests, resulting in patient harm [22]. Many healthcare services, including screening, are paid for through a fee-for-service arrangement, which can induce doctors to provide care that is not medically indicated [23–25]. What this means is that healthcare providers, who are society’s vested experts and purveyors of health, may have conflicts of interest that can manifest as “advice” recommending individuals to take a screening test [26].

Amidst the information asymmetry between doctors and laypersons, there is often an implicit trust in many healthcare contexts that the medical profession will act in the patient’s best interests and a belief that the government will intervene if screening offered is not beneficial or is harmful [27]. Furthermore, individuals may rely on heuristics rather than relevant information to make decisions, and it can be difficult to ensure that the decision to undergo screening is truly free of forms of controlling influences such as coercion and manipulation [27].

#### Inherent tension between individual interests and public benefit in screening

Screening is designed primarily to benefit the population, not the individual—most screened individuals (generally) do not have the disease of concern and will test negative, and so screening does not actually benefit their health. However, health screening is usually delivered as a clinical service within the bounds of a doctor-patient relationship in which the patient’s interests are supposed to be paramount [28]. But what is good for the population is not necessarily so for the individual—and therein lies the central conflict between promoting the best interests of individual patients and promoting the health of the population with regard to screening [3].

In providing an ethical framework for public health interventions, the Nuffield Council on Bioethics (a charitable body based in the United Kingdom that examines bioethical issues) proposes a “stewardship model” in which States “have a duty to look after important needs of people individually and collectively”. This means that States have the obligation “to provide conditions that allow people to be healthy, and, in particular, to take measures to reduce health inequalities” [29]. If we apply the stewardship model to screening programmes, it follows that States should balance individual interests (including the wish to avoid potential harms such as risk of injury, financial costs, and unnecessary disruptions to personal life) with the health benefits that potentially, only a minority of the target population will receive (which suggests that health inequalities would not be significantly reduced and healthcare resources would not be equitably distributed). It is this tension that underpins much of the debate on screening principles, and the complexities in planning and implementing screening programmes [1, 2, 30, 31].

#### Screening is not “risk-free” and potentially harmful

Although health screening is frequently cast in a favourable light when promoted to the public, screening tests are not “risk-free” as there are potential harms with their use. The possible harms from screening are driven by these main factors: (1) the accuracy of the tests; (2) the cut-offs used to distinguish between a higher and lower risk of disease; (3) and, the nature of the screening test or post-screening intervention(s).

As most screening tests are not 100% sensitive and specific, a screening result is possibly inaccurate (i.e. false positive or false negative). False positives can generate psychological distress and often result in further investigations to determine if the disease of concern is present [32]. These negative effects could be avoided if screening had not been done in the first place. With false negatives, individuals might feel reassured on receiving a “clean” bill of health when in fact they have a disease of concern [32].

“Abnormalities” are more likely to be detected as testing technology advances, giving rise to the issue of overdiagnosis; overdiagnosis has been defined as the “phenomenon of individuals receiving diagnosis (often accompanied by interventions) that, on balance, lead to greater harm than benefit” [33]. For some diseases (such as prostate, breast and thyroid cancer), a substantial proportion of cases detected through screening may never cause problems in the individual’s lifetime [32]. Another contributor to overdiagnosis is the tendency to widen definitions to include milder cases of disease [34]. For example, diagnostic thresholds for gestational diabetes were lowered in 2010 despite lack of clarity that this would lead to improved outcomes at that time, resulting in almost 1 in 5 pregnant women classified with the condition and subjecting them to the medicalisation of their pregnancies and more interventions (e.g. regular glucose monitoring) [35]. Overdiagnosis is often accompanied by overtreatment; because “disease” was detected, individuals received treatment that may not have been needed, inadvertently placing them at risk of harm from these treatments. Overtreatment also results in a waste of resources that could arguably be better used for genuine health needs [20, 32].

Interventions, whether the screening test itself or follow-up investigations or treatment, can cause harm in multiple ways—physically, psychologically, financially and socially. We will use prostate cancer as an example. Most men undergoing a prostate specific antigen (PSA) screening test will not be found to have prostate cancer—an estimated 100 cases are detected for every 1000 men aged 55 to 69 years screened over a period of 13 years. However, about 240 men would have a false positive result, which is often followed by a confirmatory prostate biopsy that carries not insignificant risks (e.g. pain, bleeding, infection). For men with prostate cancer “successfully” detected, treatment can adversely affect their quality of life—about 60% and 20% would experience sexual dysfunction and urinary incontinence, respectively [36]. While it is not possible to determine which men might have been left unharmed by the cancer if left undetected, it is likely that some would have avoided the negative impacts of the diagnostic label of prostate cancer had they not been screened.

#### Key findings in the literature on the practice of HSPs

To date, little work has been done examining the practice of HSPs. Searches were conducted in PubMed from July 2019 to February 2020 using a snowball search technique, starting with the initial search string "health screening packages"[Title] OR "executive health screening"[Title] OR "executive physicals"[Title] OR "health checkup"[Title]. Only articles in English were included. Two articles discussing the practice of HSPs in the United States were found. The first evaluated the appropriateness of executive physicals at top-ranked hospitals compared with the United States Preventive Services Task Force (USPSTF) guidelines and found that tests not recommended by the USPSTF were frequently included [16]. The author raised concerns that availability of such services at leading healthcare institutions may be perceived as an endorsement and promotion of non-recommended screening. The second was a perspective piece critiquing executive physicals as a “perfect example of what American medicine should be working to expunge: the expensive, the ineffective, and the inequitable” [7]. A third article from India evaluated 25 HSPs from eight diagnostic centres and hospitals in Mumbai, India and found that more “comprehensive” packages included tests not recommended in any guideline [37]. However, details of the HSP analysis were not provided, with the main thrust of the article being the authors’ recommendations on what should be routinely screened (in the absence of screening guidelines in India).

While not the main focus of this paper, it should be noted that there has been little to minimal evidence that HSPs or screening tests have an effect on preventing disease mortality and morbidity, though there may be some effect on surrogate outcomes (such as blood pressure control) [38–40]. What is underscored is that it is not the screening test in itself that improves health but the potential post-screening intervention that matters, and whether this intervention is effective is highly dependent on the natural course of the disease, whether individuals take it up and if they have the resources to do so.

To identify media reports in English that covered ethical issues relating to HSPs (and thereby to obtain a sense of general public awareness), a search in Google was done using the initial keywords “executive health screening packages" and “ethics” in September 2019. Subsequent searches involved substituting keywords for other terminology that HSPs may be known by (such as “health MOT”) and related ethical issues (such as “false positive” or “unnecessary testing”). The few mainstream media (newspaper and news television channel) reports identified were from Western countries with the exception of two articles (one from Singapore and the other India) [8–10, 12, 21]. They discussed potential issues with HSPs such as the generation of anxiety, unnecessary interventions and lack of appropriate follow-up. It is uncertain however what impact these reports had on individuals who read them or how it might have affected their use of HSPs.

With respect to HSPs, there is a gap in research on: (1) how these packages compare with screening principles and evidence-based recommendations; (2) how these packages are promoted and the way they present information on health risks; (3) the ethical issues in relation to these packages.

## Singapore context

Singapore is an independent city-state in South-East Asia with a population of 5.45 million in 2021, of which 3.99 million are citizens and permanent residents [41]. The Singapore healthcare system is characterised by strong government regulation and its use of markets as a policy tool to ensure quality and affordable care for citizens [42]. Healthcare is funded and delivered through a hybrid model consisting both public and private elements (government share of health expenditure was 43% in 2019) [43]. An ageing population and rising prevalence of non-communicable diseases have led to greater emphasis in recent years on preventive efforts, such as screening, to contain long-term costs in Singapore [44].

### Screening in Singapore

#### How is screening organised?

In Singapore, screening is offered by a variety of providers in both the public and private sectors. This includes primary care clinics, hospitals, community-based providers and standalone health screening companies that cater mainly to corporate clientele.

A national screening programme ‘Screen for Life’ implemented since September 2017 allows individuals to tap on subsidies for recommended screenings at private general practitioner (GP) clinics on the Community Health Assist Scheme (CHAS) [5, 45]. The CHAS enables all citizens to receive government subsidies for medical care at participating GP clinics, with higher subsidies provided for those from lower income groups [46]. The conditions screened under ‘Screen for Life’ are diabetes, hyperlipidaemia, hypertension, colorectal cancer, cervical cancer and breast cancer. Patients pay S$0 to S$5 for each screening visit, which includes the review consult with the GP. Patients are charged an additional S$25 to S$75 for mammograms but they can use MediSave (the national medical savings scheme) to pay for this. Over 100,000 individuals were screened within the first three years of the programme’s implementation, with about half of them from lower income groups [47]. While there is no publicly available information about the size of the eligible population for ‘Screen for Life’, anecdotally the uptake rate is low. Assuming all 100,000 individuals were screened for diabetes, the uptake rate for diabetes screening under ‘Screen for Life’ is estimated to be less than 10% out of an eligible population of 1.6 million (estimated based on input values used in a modelling study and a citizen population size age 40 years and above of 1.8 million) [48, 49].

Polyclinics—which provide subsidised primary care to the public—provide screening for follow-up patients but generally not for new patients due to high workloads. Community-based health screenings are also conducted by the Regional Health Systems, which are the community outreach arms of the public healthcare clusters. These are organised with community partners and are usually targeted at lower income groups to screen for cardiovascular risk factors [50–52]. Both polyclinics and the Regional Health Systems only offer recommended screening tests.

Many other screening tests not included in ‘Screen for Life’ are offered by GP clinics, standalone screening companies, and both private and public hospitals. The costs of these non-recommended tests are often borne out-of-pocket by the individual as they are not subsidised by the government and only selected private insurance plans cover regular health screenings [53]. These screening tests are often bundled together, including with recommended tests, and presented as a package.

#### How is screening regulated?

Health screening in Singapore is not tightly regulated. Clinical guidance for medical practitioners is provided by the 2019 Screening Test Review Committee report [4]. The Singapore Medical Council’s 2016 Ethical Code and Ethical Guidelines, which sets ethical standards for medical practice and the conduct of doctors, state that medical practitioners “must ensure that they [screening tests] are validated and clinically appropriate” [54]. However, neither guideline is legally enforceable and there are no regulations forbidding or restricting the use of tests lacking clinical effectiveness or cost efficiency for population screening.

The Private Hospitals and Medical Clinic Act, the key legislative tool used to regulate healthcare services in Singapore, was enacted in 1980. The healthcare landscape has significantly changed since and will be replaced by the Healthcare Services Act to strengthen governance and improve regulatory clarity. The new Act will be implemented in three phases from January 2022, with regulation of hospitals and primary care clinics, and standalone screening services (currently not regulated) to begin in the second phase in March 2023 and the third phase at end 2023, respectively. Details on what this entails are uncertain [21, 55, 56].

#### Health screening packages in Singapore

HSPs are common in Singapore and can be concurrently offered with the ‘Screen for Life’ national programme within the same GP clinic. The numbers screened under the ‘Screen for Life’ national programme are relatively small and most people likely opt for HSPs instead (which may be individual or corporate HSPs) [47]. The reason for the rather low uptake of ‘Screen for Life’ may be due to the perception that its coverage is not as “comprehensive” as a HSP [57]. Additionally, it is more lucrative for GP clinics if their patients choose a HSP over ‘Screen for Life’, and they may possibly nudge patients towards a HSP instead.

Given the widespread availability of HSPs in Singapore and the potential adverse impact of inappropriate screening, more research and analysis on this topic in the Singapore context would be important to understand the implications of the ethical issues and promote policy that supports ethical screening practices.

This study aims to understand the ethical implications of HSPs on policy and practice of screening in Singapore. The paper will: (1) describe the practice of HSPs; (2) identify the ethical issues arising from HSPs; (3) discuss the implications of these ethical issues for policymakers and healthcare providers on screening policy and practices in Singapore.

## Methodology

We conducted a content analysis of websites of providers offering general health screening packages to individuals—the clinical appropriateness, the process of undergoing a HSP and the prices of packages were analysed; websites were examined for their messages on screening. Subsequently, an analysis based on the four principles approach of beneficence, non-maleficence, autonomy and justice was used to inform the discussion of the content analysis findings and policy recommendations [28].

### Definitions

In addition to screening tests, the term “health screening” has been used to refer to screening questionnaires (e.g. PHQ-2 screening tool for depression) and the performance of a medical exam by a doctor. However, in the context of this paper, it refers to the use of investigations to identify asymptomatic individuals deemed at higher risk for disease for the purpose of further diagnostic testing and possible further treatment [1–3]. For this study, we define a general HSP as a package offered to asymptomatic individuals who do not have risk factors, other than age or sex, that would warrant further investigation and is not focused on a specific body system or area of interest (e.g. cardiovascular health, pre-marital screening). Only packages targeted at individuals were analysed as details of corporate packages were not readily available online. The HSPs sampled were general packages targeted at working-age individuals (i.e. individuals aged 20 to 64 years) in Singapore.

### Selection of HSP providers

A purposive maximum variation sampling of providers offering HSPs to individuals was conducted from July 2020 to August 2020. Providers from various sectors (i.e. public, private and social enterprises) and clinical settings (i.e. hospital and primary care) were identified. A total of 14 HSP providers’ websites on health screening were selected and analysed—five from the public sector, seven from the private sector and two social enterprises (Table 1). Five (63%) of the eight public general hospitals were selected. Six (67%) of the providers (five private and one social enterprise) included in the sample provided primary care services and together they operated 154 GP clinics, which is about 9% of GP clinics in Singapore (there are about 1,800 GP clinics in total) [58]. Three of the private providers our study altogether operated six (75%) of the eight private hospitals in Singapore [59].

**Table 1:** HSP provider characteristics

S/N	Healthcare provider	Sector	Setting^
1	Changi General Hospital	Public	Hospital (1)
2	National University Hospital	Public	Hospital (1)
3	Ng Teng Fong General Hospital	Public	Hospital (1)
4	Singapore General Hospital	Public	Hospital (1)
5	Tan Tock Seng Hospital	Public	Hospital (1)
6	AcuMed Medical Group	Private	Primary care (13 clinics)
7	Fullerton Health	Private	Primary care (12 clinics) Specialty services (does not operate hospital facility)
8	Healthway Medical Group	Private	Primary care (44 clinics)
9	Lifescan Medical Centre	Private	Standalone screening services
10	Mount Alvernia Hospital	Private (non-profit)	Hospital (1)
11	Parkway Shenton Medical Group (part of Parkway Pantai)	Private	Hospitals (4) Primary care (30 clinics)
12	Raffles Medical Group	Private	Hospital (1) Primary care (48 clinics)
13	SATA Commhealth	Social enterprise	Primary care (7 clinics) Charity healthcare services (homecare, centre-based services, mobile clinics)
14	NTUC Income	Social enterprise	Insurance provider—screening provided through partner clinics

^Number of hospitals and primary care clinics are accurate at time of submission

There are eight public general hospitals in Singapore of which three were excluded as they did not offer HSPs—a total of five public general hospitals were included in the sample. None of the 20 polyclinics (as of August 2020), which are public primary healthcare providers, offered HSPs and were excluded.

Three private providers were featured in the book “Singapore’s Health Care System: What 50 Years Have Achieved” and were included in the sample (Raffles Medical Group, Parkway Pantai and Mount Alvernia Hospital). This book documented the development of the healthcare system and was part of a series commemorating the 50th year of Singapore’s independence [60]. These three private providers (which all operated hospitals) were mentioned in the book as they played important roles in Singapore healthcare. Three non-hospital private providers mentioned in a health screening feature on blog.moneysmart.sg (a popular local financial blog) were included (one provider was excluded as there was insufficient information on their website how their HSPs were conducted) [57]. A private GP group (AcuMed Medical Group) was included as it is more representative of medium GP chains in Singapore and is well-established, having been in operation since 1973. (There is no official definition of what a medium or large GP chain is, but for this paper, a medium GP chain constitutes between two to 20 clinics and a large GP chain more than 20.) [58]

Altogether, two social enterprises were included in the sample: Singapore Anti-Tuberculosis Association (SATA) Commhealth, the only social enterprise mentioned on blog.moneysmart.sg, and the National Trades Union Congress (NTUC) [57]. NTUC is the sole national confederation of trade unions and wields significant socio-political influence in Singapore. It operates twelve social enterprises, including NTUC Income, which is an insurance cooperative (its other social enterprises include preschools, supermarkets and eldercare services) [61]. Although NTUC Income did not directly provide screening services — it does so through partner clinics, it was selected because of its wide customer base and influence on the larger insurance sector.

### Approach to ethical analysis

The four principles of beneficence, non-maleficence, autonomy and justice were used to analyse the ethical issues arising from HSPs [28]. These principles are widely regarded as foundational bioethical principles that could be used to identify and analyse ethical issues in healthcare and public health contexts. They thus serve as a useful guide in analysing the screening encounters individuals have within the context of a HSP—do the benefits outweigh the risks (beneficence and non-maleficence)? What constitutes informed consent for a HSP (autonomy)? Are HSPs an equitable use of healthcare resources (justice)?

Our policy recommendations are premised on the basis that for HSPs to be delivered in an ethical manner, it would, consistent with the four principles, require that: (1) persons benefit from health screening and it is in their best interests; (2) health screening should be conducted in a way that minimises or avoids harm; (3) persons are adequately informed on the risks and benefits of screening, and the process respects and supports their autonomy; (4) and, resources are utilised in a fair and equitable manner.

### Analysis of HSPs

The health screening web sections were analysed for elements that bear relevance to the four ethical principles: (1) clinical appropriateness of tests; (2) how HSPs are conducted; (3) HSP price range and composition; (4) messages communicated by HSP providers (Table 2).

**Table 2:** Elements of health screening web sections, guide questions and the related ethical concepts

Element from website	Guide questions	Related ethical concepts
Clinical appropriateness of screening tests in HSPs	How many tests are offered? What types of tests are offered (i.e. investigation modality)? What conditions are being screened? Are non-recommended tests being offered? (Referenced with the 2019 Screening Test Review Committee report.)	Appropriateness of tests, and their potential risks and benefits, which is relevant to issues of beneficence and non-maleficence
How HSPs are conducted (i.e. the process of a HSP)	Is there medical consultation before package selection? Is there a medical assessment included in the HSP? Is there a post-screening review with a doctor?	Were patients sufficiently appraised of the benefits, risks and alternatives of the tests (i.e. informed consent, beneficence and non-maleficence)?
Price range and composition of HSPs	How much do HSPs cost? How many HSPs does each provider offer? How do tests in the cheapest package compare with the ‘Screen for Life’ national programme? How do tests in the cheapest package compare between providers?	Is there a fair allocation of healthcare resources that is effective in promoting individual and societal welfare (i.e. justice and beneficence)?
Messages communicated by HSP providers on main page of health screening section	What are the key themes of messages communicated by HSP providers’ websites? Do the websites provide factual information about screening? Do they give a balanced view on screening (including potential harms such as false positives, overdiagnosis and overtreatment)?	Do the websites create undue anxiety or unrealistic expectations (i.e. informed consent, beneficence and non-maleficence)?

Clinical appropriateness of screening tests was assessed using the Screening Test Review Committee’s 2019 report as a reference. The Committee, which comprised experts from the Academy of Medicine Singapore (a professional and educational organisation for doctors and dentists), reviewed the evidence (namely clinical effectiveness, cost effectiveness, possible benefits and harms) and grouped tests into three categories based on their appropriateness as a screening tool (Table 3) [4]. Categorisation of investigation modalities and disease groups in this paper also referenced the Committee’s report. (Screening recommendations by the Committee are not free of the ethical issues discussed earlier but it is taken that the Committee have carefully considered the evidence supporting a test’s use or disuse in the Singapore population.) We postulated that public and non-public (private and social enterprise) providers may differ in the types of conditions they screened, and we compared the groups of conditions screened using Fisher's exact test.

**Table 3:** Categories of screening tests

Category^	Example
*Category 1—suitable for population level screening* There is good evidence that test is effective and cost efficient for population screening. A caveat is that the individual must fit the age and sex profile of the target population for the test to be considered appropriate	Fasting lipids in individuals aged 40 years and above to screen for hyperlipidaemia
*Category 2—suitable for individual level action* Test may be useful for high-risk populations or its cost effectiveness has not been evaluated or is unfavourable	Alpha-fetoprotein in hepatitis B carriers to screen for liver cancer
*Category 3—not recommended* There is insufficient evidence or the net harms outweigh benefits for use as screening test	Serum uric acid to screen for gout

^Referenced from the 2019 Screening Test Review Committee report

Coding of messages communicated on the main page of the health screening section was done by the first author. We hypothesised that providers were likely to present screening favourably, so initial coding was done using the health belief model (HBM), often used to explain screening behaviour, as a framework (Table 4) [62]. However, as not all data fit neatly into the proposed categories, Braun and Clarke’s thematic analysis approach was adopted to allow additional themes apart from those in the HBM to emerge [63]. We also analysed if providers provided a balanced view on screening and included information on downsides (such as overdiagnosis and need for further investigations). QSR NVivo 12 software was used to manage the data.

**Table 4:** Categories based on health-belief model

Health belief model construct		Application to websites featuring HSPs
Perceived susceptibility	Beliefs about susceptibility to condition	What is an individual’s risk of developing a condition to be screened?
Perceived severity	Beliefs about the seriousness of a condition	What are the consequences of a screened condition and how serious is it?
Perceived benefits	Beliefs about the effectiveness to taking action to reduce susceptibility or severity	What are the benefits of HSPs and what do they prevent?
Perceived barriers	Beliefs about the costs of taking action	How do providers address potential barriers individuals may have towards HSPs?
Cues to action	Factors that prompt action	What measures do providers use to encourage individuals to take up HSPs?
Self-efficacy	Confidence in one’s ability to take action	How do providers guide individuals through the HSP process (including follow-up of results)?

## Results

The findings of the content analysis of HSP providers’ website are presented here in the following manner: (1) the clinical appropriateness of tests in HSPs; (2) how HSPs are conducted (i.e. the process of a HSP); (3) the price range and composition of HSPs; (4) the message communicated by providers’ websites. These results provide the basis for the later discussion on ethical issues related to HSPs.

### Clinical appropriateness of tests in HSPs

#### How many tests are available?

The number of tests offered as part of a general HSP ranged from three to 71 (Table 5). All public providers offered fewer tests (range: 3–17) than private providers and social enterprises. The range of tests by private providers was 23 to 71. The number tests offered by social enterprises was 36 and 57 by NTUC Income and SATA Commhealth, respectively.

**Table 5:** Number of tests offered by providers in general HSPs

Name of provider	No. of tests^
Public	
Changi General Hospital	10
National University Hospital	3
Ng Teng Fong General Hospital	4
Singapore General Hospital	17
Tan Tock Seng Hospital	3
Private	
AcuMed Medical Group	23
Fullerton Health	42
Healthway Medical Group	59
Lifescan Medical Centre	59
Mount Alvernia Hospital	33
Parkway Shenton Medical Group	63
Raffles Medical Group	71
Social enterprise	
NTUC Income	36
SATA Commhealth	57

^These tests refer to investigations (e.g. blood, imaging) and do not include tests that are conducted as part of a physical exam (e.g. blood pressure measurement, Ishihara test for colour blindness)

#### What investigation modalities are used?

Various investigation modalities were employed by providers for HSPs, including blood, stool and imaging (Table 6). In general, private providers utilised more types of investigations compared to public hospitals. None of the public providers offered tumour markers while all the private providers and social enterprises did. Advanced imaging (CT/MRI scans) were included in general HSPs by four private providers and one social enterprise, whereas the other providers (including all five public providers) did not.

**Table 6:** Investigation modalities offered by type of providers in general HSPs

Type of provider	Investigation modality							
	Blood (non-tumour markers)	Blood (tumour markers)	Urine	Stool	Imaging (X-ray, ultrasound)	Imaging (CT/MRI)	Special^	Others*
Public (n = 5)	5	0	3	4	1	0	1	2
Private (n = 7)	7	7	7	7	7	4	6	6
Social enterprise (n = 2)	2	2	2	2	2	1	2	2
All (n = 14)	14	9	12	13	10	5	9	10

^Special tests refer to tests that require specific equipment and skills/training to administer (e.g. Pap tests, spirometry, treadmill stress test)

*Other tests refer to tests that require specific equipment and can be easily administered in the clinic setting (e.g. ECG, tonometry)

#### What conditions are HSPs screening for?

A wide range of conditions were available for general health screening (Table 7). All providers offered screening for diabetes and hyperlipidaemia, which are included under the ‘metabolic, nutritional, endocrine and rheumatology’ category. Other common categories included cancer (e.g. colorectal cancer), heart and vascular diseases (e.g. coronary heart disease), and infectious diseases (e.g. hepatitis B) for which 13, 10 and 11 providers offered testing, respectively. Less frequently tested categories were musculoskeletal (e.g. back disorder), gynaecological (e.g. menopause), and vision and hearing disorders (e.g. glaucoma). No genetic testing was offered by any of the providers. Non-public providers (private providers and social enterprises) were significantly more likely than public providers to provide tests for infectious diseases (*P* = 0.0275), gynaecology conditions (*P* = 0.031) and miscellaneous conditions (*P* = 0.0005).

**Table 7:** Groups of conditions that are screened by type of providers

Type of provider	Category^							
	Cancer	Heart and vascular diseases	Infectious diseases	Metabolic, nutritional, endocrine and rheumatology conditions	Musculoskeletal disorders	Gynaecology conditions	Vision and hearing disorders	Miscellaneous*
Public (n = 5)	4	2	2	5	0	0	0	0
Private (n = 7)	7	6	7	7	2	4	5	7
Social enterprise (n = 2)	2	2	2	2	0	2	0	2
All (n = 14)	13	10	11	14	2	6	5	9

^Categorisation of tests adapted from the 2019 Screening Test Review Committee report

*Miscellaneous conditions include benign prostatic hyperplasia and chronic obstructive pulmonary disease

#### Are non-recommended tests offered in HSPs?

Private providers and social enterprises were more likely than public providers to offer tests not recommended for population screening. All private providers and social enterprises offered Category 2 and 3 tests in their general packages compared with 60% and 40% of public providers for Category 2 and Category 3 tests (‘suitable for individual level decision’ and ‘not recommended’), respectively (Table 8). Category 1 testing was offered by all providers, although it is possible that such testing may be inappropriately performed if the patient did not fit the age profile of the target population (e.g. FIT kit testing in a 35-year-old).

**Table 8:** Type of tests included in general HSPs by type of providers

Type of provider	Category 1 (suitable for population level screening)	Category 2 (suitable for individual level action)	Category 3 (not recommended)
Public (n = 5)	5	3	2
Private (n = 7)	7	7	7
Social enterprise (n = 2)	2	2	2
All (n = 14)	14	12	11

### How HSPs are conducted

**Fig. 1:**
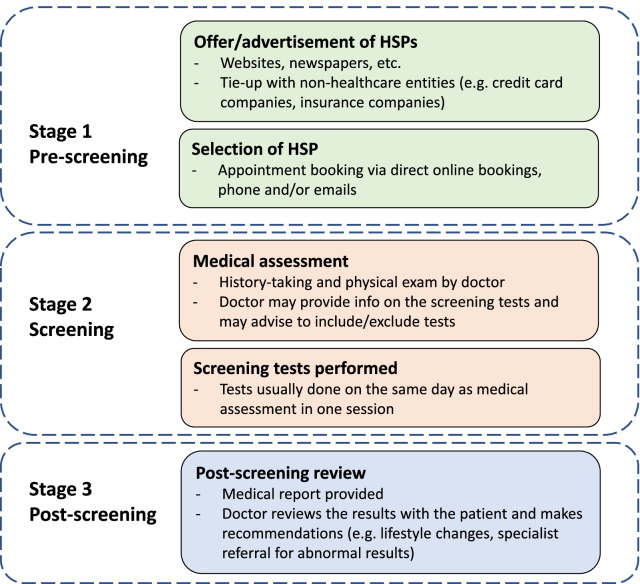
Three-stage process of a HSP

HSPs are conducted in a three-stage process—pre-screening, screening and post-screening (Fig. 1). Stage 1, the pre-screening stage, begins with an offer or advertisement of a HSP by the healthcare provider. HSPs can be advertised directly by providers through their websites or indirectly through partnerships with non-healthcare entities. Such entities include insurance companies (e.g. NTUC Income) and credit card companies (e.g. Mastercard promotion with Fullerton Health) [64, 65]. 10 providers allowed for direct online bookings (none of the public hospitals had online appointment bookings except for Ng Teng Fong General Hospital). For providers that did not have an online booking system, an appointment could be arranged via phone and/or email. None of the healthcare service providers offered a medical consultation before selection or booking of a HSP appointment.

Stage 2 starts with a medical assessment, during which a patient’s medical history is sought, and a physical examination performed. Doctors may advise on the appropriateness of screening tests during the assessment and suggest adding tests that are relevant. At the pre-screening consult, the patient would have prepped for the screening tests in their chosen HSP (e.g. fasted for diabetes and hyperlipidaemia screening) as the medical assessment often takes place right before tests are administered.

The last stage is the post-screening review, and commonly includes provision of a medical report and a consult with the doctor to review test results. During the review, screening results are explained and follow-up recommendations are made. These recommendations include advice on lifestyle changes and referrals to specialists for abnormal results for further evaluation or management. Most providers did not indicate how soon the review took place, but three providers (Changi General Hospital, Parkway Shenton Group and Fullerton Health) indicated it would be within 14 business days or 2 weeks.

### Price range and composition of HSPs

**Fig. 2:**
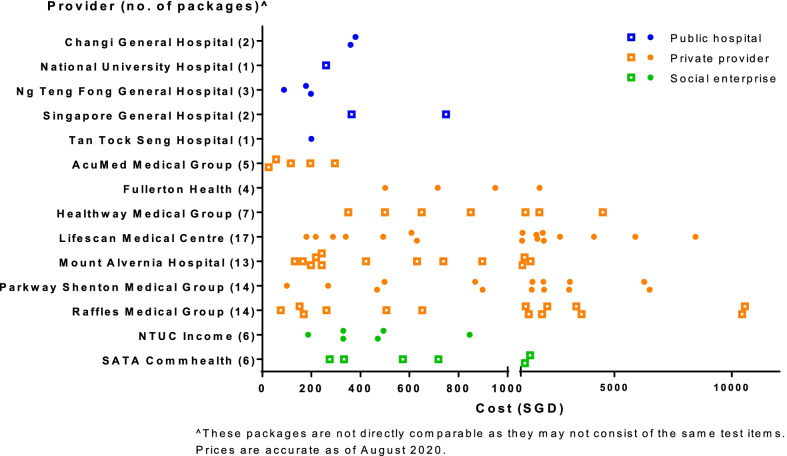
Number and price range of screening packages by provider

Public hospitals offered the fewest number of HSPs (range: 1–3) compared to private providers (range: 4–17) and social enterprises (both offered 6 HSPs) (Fig. 2). Four providers had more than 10 general screening packages that individuals could select from; all four were private providers (Raffles Medical Group, Parkway Shenton Medical Group, Mount Alvernia Hospital and Lifescan Medical Centre). There was a wide range of prices—the cheapest and most expensive packages were S$26 and S$10,561 by AcuMed Medical Group and Raffles Medical Group, respectively. Private providers had more expensive HSPs, with the packages priced S$1,500 and above all offered by private providers.

With regard to the composition of HSPs, there was considerable variation between providers, as illustrated in Table 9, which provides an overview of tests in the cheapest packages by providers. The price of the cheapest packages ranged from S$26 (AcuMed) to S$500.76 (Fullerton Health). All providers included diabetes and cholesterol screening, but differed for the rest of the tests. Other tests commonly included in the cheapest packages were urinalysis (10 providers), kidney function tests (nine providers), full blood count (nine providers), and ECG (nine providers) (Table 10).

**Table 9:** Overview of tests in cheapest packages by provider

Name of provider	Cost (S$)	Recommended tests (available under ‘Screen for Life’)^					Non-recommended tests (category 2 and 3)				
		Diabetes (fasting blood glucose)	Cholesterol (lipid panel)	Colorectal cancer (stool blood)	Cervical cancer (Pap or HPV test)	Breast cancer (mammogram)	Blood (non-tumour markers)	Blood (tumour markers)	Urine test	ECG	Chest x-ray
Public											
Changi General Hospital	360.00	✓	✓				✓		✓	✓	
National University Hospital	260.00	✓	✓						✓		
Ng Teng Fong General Hospital	88.00	✓	✓								
Singapore General Hospital	363.80	✓	✓	✓			✓		✓	✓	✓
Tan Tock Seng Hospital	200.00	✓	✓	✓							
Private											
AcuMed Medical Group	26.00	✓	✓				✓				
Fullerton Health	500.76	✓ (+ HbA1c)	✓	✓	✓		✓	✓	✓	✓	
Healthway Medical Group	350.00	✓	✓	✓	✓		✓	✓	✓	✓	✓
Lifescan Medical Centre	180.00	✓ (+ HbA1c)	✓	✓			✓	✓	✓	✓	
Mount Alvernia Hospital	131.60	✓	✓				✓			✓	
Parkway Shenton Medical Group	100.00	✓	✓				✓		✓		
Raffles Medical Group	74.90	✓	✓				✓		✓	✓	
Social enterprise											
NTUC Income	187.25	✓	✓	✓			✓		✓	✓	✓
SATA Commhealth	275.00	✓ (+ HbA1c)	✓	✓			✓		✓	✓	✓

^Hypertension screening is not included in this table as a blood pressure reading is usually obtained during the physical examination rather than an investigation

**Table 10:** Overview of category 2 and 3 tests in cheapest packages by type of provider

Test	Type of provider			
	Public (n = 5)	Private (n = 7)	Social enterprise (n = 2)	All (n = 14)
Biochemistry				
Kidney function	2	6	1	9
Liver function	2	5	1	8
Thyroid function	0	2	1	3
Calcium/ phosphate	0	4	1	5
Rheumatoid factor	0	2	0	2
Uric acid	0	6	2	8
HsCRP	0	2	0	2
Haematological				
Full blood count	2	5	2	9
ESR	0	2	0	2
Tumour markers				
AFP	0	3	0	3
CEA	0	3	0	3
PSA	0	1	0	1
CA125	0	1	0	1
Infectious disease tests				
Hepatitis A	1	3	1	5
Hepatitis B screen	2	3	2	7
Syphilis	0	1	2	3
Non-blood tests				
Urinalysis	3	5	2	10
Urine microalbumin/ creatinine ratio	0	1	0	1
ECG	2	5	2	9
Chest X-ray	1	1	2	4

For their cheapest packages, no provider offered the full suite of screenings available under the ‘Screen for Life’ national screening programme. While all providers offered diabetes and cholesterol screening, recommended cancer screenings were not included in majority of the cheapest packages—only half offered colorectal screening, two performed cervical cancer screening and none offered mammograms.

### Messages communicated by HSP websites

There were six key themes identified from the analysis of messages communicated by the providers’ main webpage on health screening (Table 11). The first informs individuals that they may be at risk due to unawareness that their lifestyle can lead to silent conditions. The second explains how screening detects conditions early and prevents serious health complications. The third describes how screening empowers an individual to act on their health. The fourth positions HSPs as a patient-centric model of care. The fifth highlights that providers will guide patients through the screening process from test selection to follow-up of results. The sixth theme presents HSPs as a premium service, with further elucidation of the following sub-themes: comprehensive screening, dedicated staff, and comfortable environment. All themes had corresponding health belief model constructs except for the sixth theme, which demonstrated how providers market HSPs as a quality service.

**Table 11:** Key themes of messages communicated by HSP providers’ webpages

Themes	Corresponding health belief model construct	Sub-themes
Individuals are at risk because of unawareness that their lifestyles can cause silent conditions	Perceived susceptibility	*“Many diseases such as diabetes, stroke and heart disease are known as silent killers, and often, the symptoms of these diseases may not be obvious.” (Tan Tock Seng Hospital)*
Screening detects conditions early and prevents serious complications	Perceived severity Perceived benefits	*“Fortunately, many of these illnesses can be detected early and treated to prevent further complications” (Changi General Hospital)*
Screening empowers individuals to act on their health and improve their well-being	Perceived benefits	*“our aim is to enhance your well-being, and help you better understand and manage your state of health.” (Raffles Medical Group)*
HSPs are positioned as patient-centric—screening services are personalised and convenient	Perceived barriers	*“we tailor health screening packages based on age, medical history, risk factors, family history and health concerns.” (Parkway Shenton Medical Group)*
Providers help guide patients through the screening process from test selection to follow-up of results	Cues to action Self-efficacy	*“A health screening doctor will accompany you throughout your screening. This allows us to answer all your health concerns as you screen with us.” (Ng Teng Fong General Hospital)*
HSP providers’ services are exclusive and top-notch: (a) Comprehensive and up-to-date screening (b) Dedicated staff (c) Comfortable environment	Nil	*“we provide personalised executive health screening in a modern and cosy environment using the latest assessment tools” (Mount Alvernia Hospital)*

Based on the websites analysed, there were no statements that would prima facie contravene the Private Hospital and Medical Clinics (Advertisement) Regulations 2019 or the Singapore Medical Council’s 2016 Ethical Code and Ethical Guidelines [54, 66]. The information provided about screening was factual, there were no exaggerated claims, and testimonies were not used. While the potential benefits of screening and HSPs were discussed (as reflected in the thematic analysis), none of the providers provided information about screening that would be construed as negative—there was a lack of information about clinical effectiveness of tests, and potential risks and harms of screening (such as false positives or false negatives, overdiagnosis, the possible identification of incidentalomas or the potential complications of more invasive investigations).

## Discussion

This study explored how HSPs targeted at individuals were conducted and promoted, how clinically appropriate HSP tests were, and the price range of packages. Most HSP providers, particularly private providers and social enterprises, included tests that were inappropriate for population screening. Apart from diabetes and cholesterol screening, there were otherwise no “standard” tests in HSPs. There was a diversity of test modalities and conditions being screened, which was reflected in the huge price range of HSPs from S$26 to S$10,561, with private providers having more expensive HSPs on offer. However, none of the cheapest packages (even for private providers) included all tests recommended under the ‘Screen for Life’ programme.

HSPs can be selected and booked with little to no medical guidance, even though more complex tests such as advanced imaging and endoscopic procedures such as nasoendoscopy were on offer. All providers in this study offered a medical assessment and a post-screening review to explain results. The websites presented HSPs positively as means of preventing complications through early detection and promoted providers’ services as being of good quality. They did not however discuss negative aspects of HSPs, such as direct risks from tests (e.g. risk of bleeding with a gastroscopy) or the possibility of false positives or false negatives.

### Ethical issues arising from health screening packages

Despite the widespread availability of HSPs, they may not be in patients’ best interests and current practices raise many ethical questions. The inclusion of non-recommended tests may lead to an unfavourable risk–benefit ratio and the deluge of tests on offer can make it difficult for patients to be sufficiently apprised to make an informed decision. Furthermore, HSPs may result in a maldistribution of resources as scarce healthcare resources are funnelled towards screening which may not be beneficial or which exposes patients to unnecessary harms.

#### Beneficence and non-maleficence

Much of the debate and work on screening principles is based on the criteria Wilson and Jungner outlined in their seminal text ‘Principles and Practice of Screening for Disease’ [1, 2, 30, 31]. Although these criteria do not explicitly discuss ethical principles, beneficence and non-maleficence are clearly embedded within them. Many tests offered in the HSPs analysed are worryingly not aligned with screening principles. While it is unsurprising that all private providers and social enterprises offered non-recommended (Category 2 and 3) tests in their packages given the commercial interest to do so, it is concerning that 60% of the public providers sampled offered such tests, given the ethos of serving the public good that is generally associated with this sector. It does seem to suggest that the use of non-recommended tests in HSPs, which are more likely to be of minimal benefit and higher risk, may be pervasive in Singapore across all sectors.

Furthermore, some of the cheapest “basic” packages do not include screenings that are actually recommended. An example is cervical cancer screening (HPV DNA or Pap test depending on age). This recommended test was included in the cheapest packages by only two of the providers studied, and was sometimes offered as an add-on rather than included in the main package. A possible reason could be that the test requires additional skill, preparation and time to perform, whereas non-recommended tests (e.g. kidney function tests) can be done on the same sample collected for other recommended tests (e.g. fasting lipids). What this suggests is that HSPs may be designed more towards adding business value for providers rather than ensuring that patients get their recommended screenings.

Most providers analysed offered non-recommended screening (12 and 11 providers offered category 2 and 3 tests, respectively)—the use of tests lacking evidence for population screening increases the exposure of individuals to a litany of harms such as false positives or negatives, need for further investigations (that may be invasive), overdiagnosis and overtreatment, psychological distress and financial costs [3, 20, 32, 33]. However, none of the providers’ websites communicated these harms. Instead, these websites promoted the extensive use of these tests as a “comprehensive” assessment that empowers individuals to act on their health, which are not supported by beneficence and non-maleficence.

The lack of appropriate follow-up post-screening has also been surfaced in media reports, which have highlighted issues that could result from this, such as potential confusion over results or a missed opportunity for early treatment [8, 21]. This did not appear to be an issue for the HSPs analysed as all providers offered a medical consult post-screening. A possible reason could be that these packages were targeted at individuals and costed more compared to corporate packages (which may be discounted), and hence included more services such as a post-screening consult.

#### Autonomy and informed consent

Autonomy can be defined as “a notion that relates to how well a person is able to control her life according to her own decisions and actions” [3]. Informed consent, which is predicated on respect for autonomy, requires that a patient has adequate understanding of relevant and sufficient information in order to make a decision [28]. On the surface, it may appear that HSP providers in our sample respect or even promote autonomy by providing individuals information on their health status and personalised recommendations that they can act upon to improve health. However, a deeper dive on the HSP process shows that the converse is true. What would respect for autonomy look like for HSPs? Essentially, the individual should be provided balanced information about screening that would enable them to make an informed decision that is in their best interests, and the process to do so should be free from controlling influences such as manipulation.

While all websites in this study explained the benefits of screening and provided information on the process, there was virtually no information about potential harms. This absence of information is likely to be compounded by a lack of public understanding in Singapore about the benefits and risks of HSPs; an example is how financial blogs presented HSPs positively as a “comprehensive” health check without mention of potential harms [53, 57]. Such favourable views on HSPs by laypersons may be reinforced by silence from health authorities on HSPs. The Health Promotion Board, the statutory board responsible for health promotion in Singapore, briefly mentions that some tests may not be recommended and provides a link to the 2019 Report of the Screening Test Review Committee (which is intended for a medical audience) [67]. It does not however provide material pitched to the general public that enables them to better navigate HSPs and their appropriate utilisation.

Within the context of an information gap and the multitude of tests on offer (up to 71 tests for one provider analysed), it would be unreasonable to expect the average layperson to have adequate grasp of the topic to make an informed selection when experts themselves may not even agree if a particular test should be recommended [33]. Furthermore, even if detailed information on the pros and cons of each screening test were made available on providers’ websites, it does not necessarily mean that the individual’s decision-making process is free from manipulation. Because individual decision-making may be based on heuristics rather than relevant information, and because of the societal trust enjoyed by healthcare providers, laypersons may assume that providers are offering these tests because they are beneficial for health and proceed with them without actively seeking further information [27]. Additionally, healthcare providers may offer financial incentives to attract individuals to undergo a HSP, such as one provider in our study (Fullerton Health’s partnership with Mastercard, a credit card company) [65]. It is probable that such commercial inducements have an effect on individual screening decisions, which may not be purely based on “clinical” factors.

All the providers analysed allowed for package selection before a medical consult. This has similarities with issues faced in direct-to-consumer genetic testing in which consumers may be misled about the analytic and clinical validity of tests [68]. Arguably, doctors could discuss with patients on the risks and benefits of each test listed in the HSP during the consult before tests are administered; however, such an attempt (even if feasible) would not constitute informed consent—this may be perceived as “information dumping”, which inhibits understanding and thus undermines autonomy [3]. Permitting the selection of a HSP before a medical consult is also in conflict to a statement in the 2016 Singapore Medical Council Handbook on Medical Ethics—a resource which expounds on what the 2016 Ethical Code and Guidelines mean and provides advice on best practices—that doctors should “[t]ake a detailed history and perform a thorough clinical examination to detect health risks and plan appropriate screening tests” [69]. It also runs counter to providers’ claims that they provide “personalised” screening.

Our study showed that providers charged higher when more tests were performed, and there may be a potential conflict of interest for doctors between giving objective medical advice and encouraging more tests to improve earnings. Also, the most expensive package (S$10,561) analysed was more than 100 times the cost of recommended screenings under ‘Screen for Life’ (the most one may pay is S$80, inclusive of mammogram). In light of other studies showing the impact that financial incentives can have on medical care or advice rendered, it is reasonable to assume that doctors offering HSPs in our study may have strong extraneous interests that could influence information provided to patients that affects a patient’s ability to make an informed decision [22, 26].

#### Justice

This study found that HSP providers can charge patients hundreds to thousands of dollars for screening tests that may be unnecessary and potentially even harmful. Compared to the S$0 to S$5 fee that patients pay for recommended screenings and additional $25 to $75 for a mammogram under ‘Screen for Life’, HSPs may be pricier and much less “value-for-money” than the national screening programme. HSPs appear to allow scarce healthcare resources (e.g. advanced imaging) to be made available based on who is willing to pay rather than according to clinical need, potentially resulting in a maldistribution of resources and worsening inequities.

Patients who undergo HSPs and receive abnormal results may be advised on further testing and/or follow-up with a specialist. In some instances, this additional healthcare utilisation could have been avoided if the patient had not chosen a HSP to begin with. Often these abnormal findings are benign, but may set off a cascade of anxiety, additional investigations and follow-ups that may not be warranted in the first place [20]. An example is a thyroid nodule detected on ultrasound (which was offered by four of the providers in our study)—although majority of these lesions are benign, the small possibility that they are cancerous may necessitate regular follow-up to monitor their size [70]. Moreover, there is anecdotal evidence that some patients who underwent a HSP in the private sector and had an abnormal result will obtain a subsidised referral at the polyclinic to follow up with a specialist in the public sector. HSPs can potentially create additional demand for limited resources for care that may not be clinically necessary or cost-effective at the system level.

In our study, we identified five public hospitals and two social enterprises who offered HSPs. One possible reason for this is that profits earned from these packages may be used to off-set costs for other healthcare services that bring in less revenue. However, such reasoning is not ethically justifiable as these HSPs result in diversion of scarce resources away from other needs in the hospital and at a system level. Rather than using their resources to improve patient and population health through effective interventions, it is being used to stimulate unnecessary demand for healthcare testing through HSPs. For example, none of the public and social enterprise providers offered cervical and breast cancer screening in their cheapest packages even though these tests are recommended for population level screening.

### Policy recommendations

The ethical issues discussed are complex and measures to address them would have to consider the health system at large. Health screening is not solely a population health intervention; it is a commercial activity in which the relationship between doctor and patient may also be characterised as one between buyer and seller. However, such a consumer-centric approach to screening can result in providers being incentivised to include more tests (which are often inappropriate) in HSPs. Instead, the guiding principles for this paper’s recommendations draw from the four principles approach to biomedical ethics: (1) persons should benefit from health screening, and harms should be minimised or avoided; (2) persons are adequately informed on the risks, benefits and alternatives of screening so as to respect their autonomy; (3) resources should be distributed according to need rather than ability to pay.

Additionally, policy recommendations should be feasible to implement and the intrusiveness of interventions should be justified by the public health benefits [29]. While it may be possible to legislate a ban on the use of non-recommended screening tests, such a blunt measure would be impractical and possibly excessive.

Based on the study findings, we propose recommendations to: (1) educate the public about screening and HSPs to reduce the information gap; (2) tighten regulations to minimise the use of tests not recommended for population screening, such as limiting advertisement of these tests; (3) limit the provision of screening services in hospitals to promote appropriate allocation of healthcare resources.

#### Public education on screening

First, there should be better public education on screening by health authorities such as the Health Promotion Board. As it would be impractical to require individuals to attend pre-screening consults for all tests, a more feasible approach would be improving the general public’s awareness. Current public engagement is focused on promoting the national screening programme and its benefits, with minimal guidance on non-recommended tests [5, 67]. More education should be provided on non-recommended testing, including potential risks. Not doing so may undermine national screening efforts as it can give rise to false perceptions that the ‘Screen for Life’ programme is incomplete or inadequate [53, 57]. We acknowledge that the provision of information may not translate into better public understanding on the benefits and risks of screening and HSPs as there can be many other factors such as systems and structural factors affecting an individual’s decision-making. However, there is presently no such information targeted to the general public from health authorities or advocacy groups in Singapore, and making available such information would be a necessary first step towards addressing the information asymmetry between providers and laypersons.

#### Tightening regulations on general population screening

Second, regulations on what type of tests can be offered for general population screening should be tightened and the impending regulation of health screening services under the Healthcare Services Act provides an opportunity to do so. These regulations should apply to all health screening (including corporate packages), not just standalone screening services. Providers should not be allowed to advertise or offer non-recommended tests as general screening at the first instance. Non-recommended screening may still be performed, but only after a medical consultation during which the patient is properly apprised of the benefits, risks and alternatives of such tests. A post-screening review with a healthcare professional should be offered, even for normal results, as patients may have queries related to the results or seek advice on preventive activities. Review for abnormal results should be done with a doctor to ensure that the patient is properly counselled and advised on them.

#### Limiting the provision of HSPs in hospitals

Lastly, the provision of HSPs in hospitals should be limited. Screening services should ideally be sited within primary care, where a regular provider oversees and coordinates a patient’s care. Majority of screening tests, including those in ‘Screen for Life’, do not require tertiary level provision and may create unnecessary demand for hospital resources. Such services may contribute to the misperception that hospital screenings are of a higher quality than those done in primary care, and may perpetuate inequities through its promotion of a model of care based on the ability to pay rather than on patients’ needs. For public hospitals, limiting its provision of HSP services could be done through the Ministry of Health’s 'level of medical capability’ framework, which determines what level of service capabilities (and resourcing) each hospital should provide [71]. Medically indicated or opportunistic screenings may be allowed, but there should not be a clinical service dedicated to general health screening in the hospital. However, this framework does not apply to private hospitals for which the only way to implement this measure is through legislation—again, this is possibly excessive. Instead, better information on HSPs from health authorities and advertising restrictions as described earlier may be sufficiently effective to curb provider-induced demand in the non-public sector.

### Strengths and limitations

The strength of this study is that it provides a detailed description of how HSPs are conducted in Singapore, how tests compare with evidence-based recommendations, how much patients are charged for such testing, and how HSPs are being promoted (including what messages they convey about screening) where such knowledge is currently lacking. While the scientific aspects of screening have been examined before, this is the first study that highlights the key ethical issues arising from the practice of HSPs and explores the implications of these issues on screening policy and practice in Singapore, adding another dimension of understanding to the literature on screening complexities.

The main limitation of this study was that purposive sampling was employed and it did not examine corporate HSPs. Only providers with readily available information on their websites were included in the study (this was generally more of an issue for corporate HSPs). While information on HSPs could have been sought from channels such as through phone calls or emails, this was not feasible during the conduct of the study due to logistical and time constraints. As such, the findings of this study may not be representative nor generalisable to all healthcare organisations providing HSPs. Additionally, the study did not include the perspectives of patients who underwent HSPs or doctors who provided such screening—these views would have been useful to better understand why patients went for such tests, what the perceived ethical issues are, and the implications for screening behaviour. Coding of messages communicated on the main page of the health screening section was done by a single coder, which may have resulted in failure to recognise other possible themes. Future research that takes a more representative sample, including corporate HSPs, would be useful. In addition, seeking provider and patient perceptions on HSPs and screening in general would contribute to better understanding of their decision-making process and allow for a more informed health screening policy.

## Conclusion

In conclusion, this paper identifies several ethical issues that should be addressed with regard to HSPs in Singapore. The information gap between providers and patients is a significant area that policy changes can help address, so that patients are better informed about the HSPs they might undertake. The use of non-recommended screening tests should be better regulated, to protect the interests of patients and to move from a consumer-oriented health system to one that promotes population health and equitable use of scarce medical resources.

## Data Availability

The datasets used and/or analysed during the current study available from the corresponding author on reasonable request.

## Abbreviations

CHAS: Community Health Assist Scheme
CT: Computed tomography
ECG: Electrocardiogram
ESR: Erythrocyte sedimentation rate
FIT: Faecal immunochemical test
GP: General practitioner
HBM: Health belief model
HPV DNA: Human papilloma virus deoxyribonucleic acid
HsCRP: High-sensitivity C-reactive protein
HSP: Health screening package
MOT: Measurements, observations and tests
MRI: Magnetic resonance imaging
NTUC: National Trades Union Congress
PHQ-2: Patient Health Questionnaire-2
PSA: Prostate-specific antigen
SATA: Singapore Anti-Tuberculosis Association
USPSTF: United States Preventive Services Task Force
WHO: World Health Organization
